# The Extraction of Children’s Teeth

**Published:** 1862-08

**Authors:** W. H. Atkinson


### "THE EXTRACTION OF CHILDREN'S TEETH."

BY DR. W. H. ATKINSON.

Read before the N. Y. Society of Dental Surgeons, May 28, 1862.

THIS is a painful subject for contemplation to the humane mind; not only because of the sorry neglect that renders it ever necessary, but also from its very frequent occurrence in even enlightened and otherwise Christian-like human hands.

There are two distinct classes in which it never becomes necessary, or is ever thought of but as the rarest of unpleasant interference with nature's useful processes, viz., the purely natural specimen of instructive living in savage life and the truly cultivated class who have attained sufficient knowledge to obey the indications of nature by a proper mode of life, in accordance with the law of development, in giving full and free exercise to the whole system, at regular intervals of activity and rest.

The representatives of these two classes are, in civilized society I am painfully aware, few and scattered in our day to such extent as to afford us little more than cause enough to prove the truth of the doctrine which they indicate as the law of the procedure of the plan of organization.

In the earlier times, philosophers and philanthropists gave almost exclusive attention to physical development and culture, to the apparent neglect of the higher moral and scholastic exercises of the brain and nervous system.

And as if to offset and balance this neglect of moral and mental in favor of the physical ability, the moderns have so stimulated the mental, I will not say the moral, that the vital pabulum has nearly all been invited to the brain and nervous system, rendering the muscular and osseous frail and attenuated to such extent as to call now loudly upon all who deprecate an absolute degeneracy of the race, to bestir themselves in favor of the proper culture of the whole man.

To such an alarming extent has this gone, that in the United States of America particularly, the cities have to depend upon an influx of country raised lads and lasses to populate the places left vacant by the dead and dying progeny of the well-to-do citizens, who live to pamper appetite and passion, rather than to improve the world by living in it fully up to the law of moral, mental and physical being.

The nutrition of the hard parts being under the law of low and uncomplicated affinities is first to suffer from departure from the proper regimen, and hence we have poorly formed and illy developed teeth as the rule in the great majority of children in the middle classes in cities.

And unfortunately, these are the very persons who have neither the time, means nor disposition to give their children's teeth the attention they demand, until, by being disturbed from their rest or their labors or pleasures by their cries, they are induced to give some attention to them, which usually results in some soothing application for a few times, and then in a fit of impatience, a rapid visit to some pliant "tooth extractor," with the imperative demand to "take that tooth (or teeth) out!!" To which, I am sorry to say, that they in the main meet with a too ready compliance, instead of a dispassionate, careful and full survey of the case, and the firm but kindly spoken opinion that the tooth is not "ready to come out;" from which opinion, or practice in accordance with it, no bullying or threatening to "go to some one who will be glad to do it," can make them swerve.

No professional man, worthy of his calling, will allow himself to be dictated to as to the propriety or impropriety of an operation by the assurance of the ignorant patient, parent or guardian that they "will take the responsibility." No one can take the responsibility from the shoulders of him who is weak enough to violate his own sense of right and proper procedure in the case for the sake of fee or favor of those who, for the time, seem to hold both his living and reputation within their power.

Extracting children's teeth is a species of murder that, like other forms of homicide, "will out," in spite of all efforts to cover up the misdeeds of ignorance and impatience!

Does any dentist ask what he shall do in such cases? I reply, do just as you would in any other instance of opportunity to do an unclean act for unclean gain! at least refuse yourself to be privy to the misdeed.

I blush to know that the weak excuse, "if I do not do it, some one else will, and I may as well have the fee now, while it is in my power," has sufficed to quiet the very feeble prick of the remains of a conscience with which some persons, I will not say men, manage to get along at a poor blundering, dying rate. And on this and like slender excuse for the sin, untold numbers of innocents are fractionalized and distorted, because there are those in places of responsibility who have thus practically said, "Evil, be thou my good." And have thus given ample opportunity to demonstrate the desirableness of a complete organization by multiplying its opposite, in the distortion of the sweet faces we meet at every turn in our walks where human beings most do congregate.

A close scrutiny of the forms of faces in the communities of children will disclose to us the unwelcome fact that a very small minority of them have regular and fully developed jaws and teeth; not over five per cent. enjoying that blessing at sixteen years of age.

How shall we bring about a better state of things? By taking more care of the dear hopes of the world at the *proper time*, which, alas! has been so universally adjudged "entirely too soon to be of any service!"

The great anxiety of parent and dentist would seem to have been to conspire in seeing by what means these "pests of childhood and nurses" could be soonest got rid of without ever seeming to think that their premature removal could in any way interfere with the perfection of the permanent teeth with which the dear mammas were *so* anxious their daughters at least, should enter the drawing-room, to dazzle all beholders by the matchless perfection of face and form, which *now*

is acknowledged not to be complete without a perfect set of clean and well arranged TEETH!!

If these things be so (and they are undeniable), when should we extract children's teeth?

*Ans.* When we can pick them off the gums with our fingers or a bit of common thread, made into a slip-noose. Save them till then by any and by all means, and be assured you nor your patients will ever have cause to regret it.

NEW YORK, May 27, 1862.

